# Community-acquired bacteremic *Streptomyces atratus* pneumonia in animmunocompetent adult: a case report

**DOI:** 10.1186/s13256-015-0753-y

**Published:** 2015-11-20

**Authors:** Miguel Angel Ariza-Prota, Ana Pando-Sandoval, David Fole-Vázquez, Marta García-Clemente, Teresa Budiño, Pere Casan

**Affiliations:** Hospital Universitario Central de Asturias (HUCA), Instituto Nacional de Silicosis (INS), Área del Pulmón, Facultad de Medicina, Universidad de Oviedo, Avenida Roma s/n, Oviedo, Asturias 33011 Spain

**Keywords:** Bacteremia, Diagnosis, Infection, Pathogen, Pneumonia, *Streptomyces*

## Abstract

**Introduction:**

*Streptomyces* spp. are aerobic, Gram-positive bacteria of the order Actinomycetales, known for their ability to produce antimicrobial molecules such as streptomycin. Pneumonia due to *Streptomyces* is considered to be rare and limited to immunocompromised patients. *Streptomyces* spp. are only rarely associated with invasive systemic infections. To our knowledge, we report the first documented case of community-acquired *Streptomyces atratus* bacteremic pneumonia in an immunocompetent patient.

**Case presentation:**

We describe a case of *Streptomyces atratus* bacteremic pneumonia in an otherwise healthy, 77-year-old Spanish man. *Streptomyces* identified by 16S ribosomal RNA sequencing grew in multiple blood cultures and bronchoalveolar lavage cultures. The infection resolved completely after treatment with imipenem and amoxicillin/clavulanic acid for 2 months.

**Conclusions:**

The majority of cases reported in the literature make reference to the difficulty of determining the pathogenic role of *Streptomyces* spp. Usually considered a contaminant, the pathogenic role of *Streptomyces* spp. is easier to confirm when the species is isolated from a catheter tip and, in the case of blood cultures, in more than one sample with a high count of colonies. To our knowledge, we report the first documented case of *Streptomyces atratus* bacteremic pneumonia in an immunocompetent patient. As the experience is limited, further studies are needed to better understand the interpretation of the isolates of the genus *Streptomyces*; the predisposing factors for infection; and the course, treatment, and evolution of these infections.

## Introduction

*Streptomyces* spp. are Gram-positive aerobic bacteria that tend to form branches and filaments. The genus *Streptomyces* belongs to the order Actinomycetales, along with human pathogens of the genera *Nocardia*, *Actinomyces*, *Dermatophilus*, and *Mycobacterium* [1]. The development of molecular biological tools such as 16 ribosomal RNA (rRNA) gene sequencing has led to modifications in the methods of identification. The molecular identification is rapid, and, in most cases, the results are unequivocal [2]. Pneumonia due to *Streptomyces* is considered to be rare and limited to immunocompromised patients. *Streptomyces* spp. are only rarely associated with invasive systemic infections [3]. To our knowledge, we report the first documented case of *Streptomyces atratus* bacteremic pneumonia in an immunocompetent patient.

## Case presentation

A 77-year-old Spanish man was admitted to our hospital in September 2012 after 10 days of marked general syndrome (asthenia, hyporexia, and 2-kg weight loss) accompanied by cough and mucopurulent sputum, dyspnea, and low-grade fever. He had a 20 pack-year history of smoking. He had no surgical background or other medical background of interest.

The clinical findings were as follows: body temperature 38.9 °C, blood pressure 170/80 mmHg, heart rate 110 beats/min, respiratory rate 30 breaths/min, and oxygen saturation 72 % on room air. The patient’s physical examination was normal except for pulmonary auscultation. Diminished respiratory sounds and crackles were found bilaterally at the bases of both lungs. Laboratory tests revealed a 20,300 × 10^9^/L white blood cell count with 90 % neutrophils, 13.1 g/dl hemoglobin, C-reactive protein 21.7 mg/L, procalcitonin (PCT) 2.84 ng/ml, and glucose 246 mg/dl. The patient’s platelet count, arterial blood clotting, and the rest of the biochemical tests were within normal ranges. Arterial blood gas analysis showed partial pressure of arterial oxygen 32 mmHg, partial pressure of arterial carbon dioxide 76 mmHg, pH 7.37, and standard HCO_3_ 35 mEq/L (room air).

**Fig. 1:**
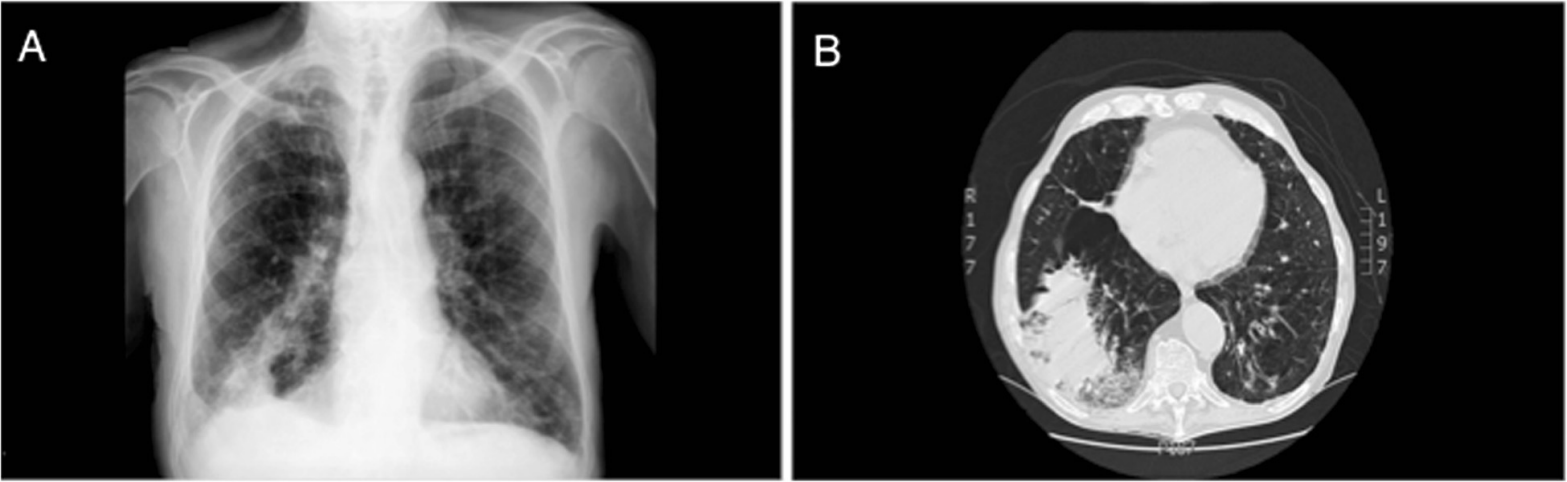
**a** Posteroanterior chest x-ray of the right lower lobe with alveolar infiltrate and a reticular interstitial pattern at the lung bases. **b** Chest computed tomographic scan showing right lower lobe alveolar consolidation with minimal pleural effusion, severe bilateral diffuse emphysema, and signs of chronic bronchitis.

A chest x-ray revealed a right lung basal alveolar infiltrate and a reticular interstitial pattern at the lung bases (Fig. 1a). The results of urinary antigen for *Pneumococcus* and *Legionella*, sputum cytology, mycobacterial culture, and serologic HIV tests were negative. Three blood cultures were obtained upon admission from the same site through a single venipuncture, and antibiotic treatment with levofloxacin was initiated. Chest computed tomography (CT) was performed 2 days after admission because of persistence of fever and radiological worsening. Levofloxacin was replaced by a broad-spectrum antibiotic (imipenem) on the third day of hospitalization. The CT scan showed a right lower lobe alveolar consolidation with minimal pleural effusion, severe bilateral diffuse emphysema, and signs of chronic bronchitis (Fig. 1b). A bronchoscopy with bronchoalveolar lavage (BAL) was performed. The quantitative cultures of BAL specimens yielded a Gram-positive aerobic branched, nonfragmented bacillus at a concentration of 4 × 10^6^ colony-forming units per milliliter identified as *S. atratus*. No other pathogen was isolated. The microbiology department confirmed *S. atratus* growth in two blood cultures on the basis of 16S rRNA gene sequencing. The isolate was sensitive to amoxicillin-clavulanic acid, erythromycin, cefotaxime, imipenem, gentamicin, tobramycin, ciprofloxacin, co-trimoxazole, clarithromycin, and linezolid.

**Fig. 2:**
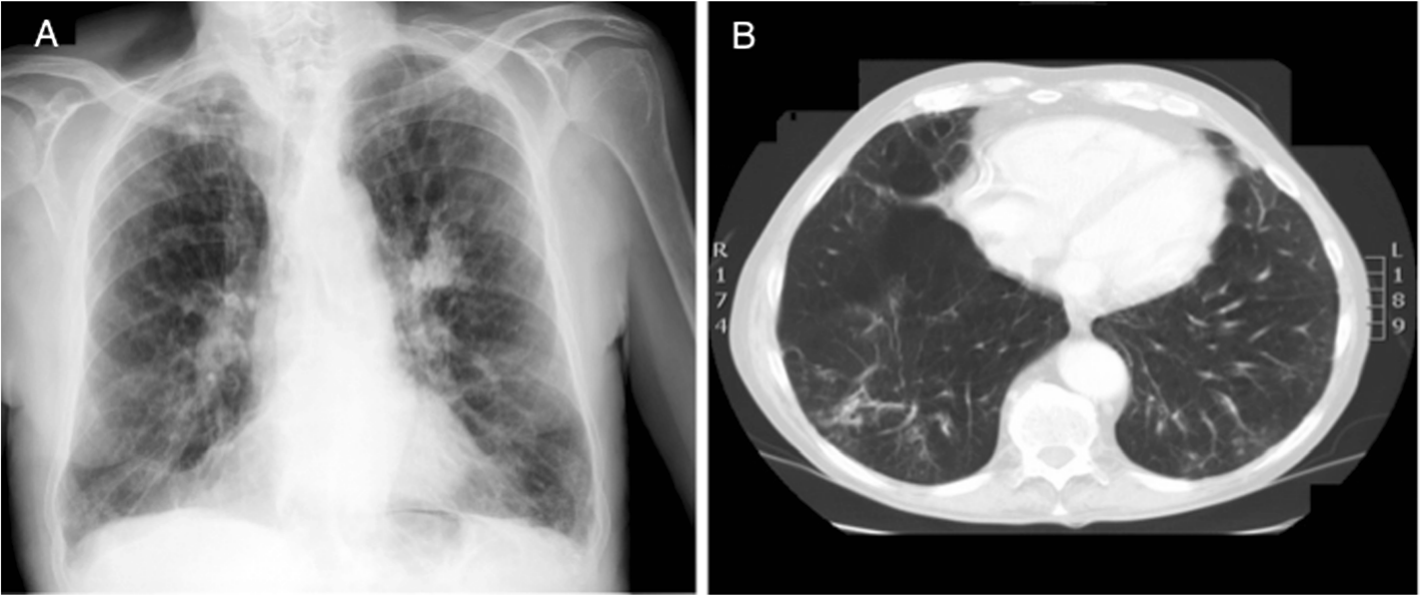
**a** Posteroanterior chest x-ray showing almost complete resolution of the right lower lobe alveolar infiltrate. **b** Chest computed tomographic scan showing almost complete resolution of the right lower lobe consolidation.

A control chest x-ray obtained 3 weeks later showed radiological improvement (Fig. 2a). The patient was treated with intravenous imipenem (2 g/day for 14 days) and changed to oral amoxicillin-clavulanic acid (1000/62.5 mg twice daily for 21 days). After 2 weeks of treatment, the patient’s blood cultures were negative and his PCT level was 0.10 ng/ml. On the basis of his clinical and radiological improvement, the patient was discharged. A control CT scan of the chest obtained at the 3-month follow-up visit showed almost complete resolution of the right lower lobe consolidation (Fig. 2b). The patient remained well at the 4-month and 6-month follow-up visits.

## Discussion

*Streptomyces* spp. comprise Gram-positive, extensively branched, filamentous bacteria that form aerial hyphae with chains of spores [1]. The streptomycetes are classified as a separate genus within the aerobic actinomycetes and are most well known for the many antimicrobial substances isolated from the approximately 600 different species [1]. With the exception of specimens from actinomycotic mycetoma, the isolation of *Streptomyces* from clinical specimens is frequently considered laboratory contamination [1]. Rare cases of clinical disease have been published; however, in almost all of these cases, there was an associated immunosuppression condition, and *Streptomyces* was not the only potential pathogen isolated. Our patient was immunocompetent, and *Streptomyces atratus* was the only isolated pathogen in two blood cultures and in the BAL culture.

Clinical situations such as host immune status, location and chronicity of infection, and response to treatment will likely dictate the intervention approach [3]. Invasive *Streptomyces* infections most often occur in immunocompromised patients with human HIV infection or malignancy and in those treated with immunosuppressive agents such as corticosteroids and cancer chemotherapy [4]. The selection of antibiotic for the treatment of individual cases should be based on the *in vitro* antibiotic susceptibility of the isolate [5]. Our patient was empirically first treated with levofloxacin for 2 days, but because the patient’s fever persisted his therapy was modified to a broad-spectrum antibiotic (imipenem). The blood cultures and the BAL culture confirmed *Streptomyces atratus* species with antibiotic susceptibility to amoxicillin-clavulanic acid, cefotaxime, imipenem, gentamicin, tobramycin, ciprofloxacin, co-trimoxazole, clarithromycin, and linezolid and without any resistance to other antibiotics. Our patient was treated for the first 3 days with levofloxacin, but his treatment was modified to imipenem because of persistent fever. He responded well to treatment with imipenem and afterward to oral amoxicillin-clavulanic acid.

We used PubMed (http://www.ncbi.nlm.nih.gov/PubMed/) to search for literature on *Streptomyces* spp. bacteremic pneumonia with the following index term: “*Streptomyces* spp. bacteremic pneumonia.” A total of only three clinical cases were found in the search. Only one of these involved an immunocompetent patient, and the case was due to *Streptomyces lanatus* species; the other two cases were in immunocompromised patients. In contrast, our patient was immunocompetent, and the use of inhaled corticosteroids in moderate doses because of his COPD should not be considered as immunosuppressive, this makes our case even rarer and interesting due to its almost zero frequency. This makes our case even rarer and more interesting owing to its nearly nonexistent occurrence. We also searched the literature for reported cases of *Streptomyces* spp. nonbacteremic pneumonia and found only seven cases. In most of these cases, AIDS, advanced malignancies, or other serious diseases that required treatment with immunosuppressives were present. Other reported cases of invasive *Streptomyces* infections include endocarditis of the prosthetic valve, catheter-related bacteremia in a patient receiving holistic infusions, bacteremia with thrombosis, pneumonia, pericarditis, peritonitis, arthritis, cervical lymphadenitis, brain abscess, and intraspinal mycetoma [6, 7]. In all the reported cases of bacteremia due to *Streptomyces*, the source was a catheter, prosthetic valve, intravenous infusion, or primary lesion in internal organs such as the lung.

A study of *in vitro* susceptibility of ten *Streptomyces* strains revealed rifampin, erythromycin, tobramycin, fusidic acid, and streptomycin sulfate as the most powerful antibiotics, whereas trimethoprim-sulfamethoxazole (TMP-SMX) seemed ineffective [8]. The most potent drugs were minocycline, imipenem, erythromycin, doxycycline, and aminoglycosides, whereas a significant percentage of resistance to TMP-SMX, ampicillin, and ciprofloxacin was noted [9]. This suggests that, in contrast to nocardiosis, TMP-SMX is not the drug of choice for *Streptomyces*. The fact that *Streptomyces* spp. are slow-growing bacteria renders prolongation of antibacterial treatment necessary [10]. In our patient, the *S. atratus* isolate was not resistant to any of the tested antibiotics.

In another investigation of BAL specimens from 247 immunocompromised patients, among others, 1 strain of *N. asteroides* and 2 strains of *Streptomyces* spp. were isolated [11]. An immunosuppressive condition was always associated in all the reported cases where *Streptomyces* spp. grew in BAL culture. Our patient was immunocompetent.

## Conclusion

The majority of cases reported in the literature make reference to the difficulty of determining the pathogenic role of *Streptomyces* spp. because it is usually considered as a contaminant. It is easier to confirm its pathogenic condition when it is isolated from a catheter tip and, in the case of blood cultures, in more than one sample with a high count of colonies. To the best of our knowledge, we report the first documented case of community-acquired *Streptomyces atratus* bacteremic pneumonia in an immunocompetent patient. As the experience is limited, further studies are needed to better understand the interpretation of the isolates of the genus *Streptomyces*; the predisposing factors for infection; and the course, treatment, and evolution of these infections.

## Consent

Written informed consent was obtained from the patient for publication of this case report and accompanying images. A copy of the written consent is available for review by the Editor-in-Chief of this journal.

## Abbreviations

BAL: bronchoalveolar lavage
CT: computed tomography
HTN: hypertension
PCT: procalcitonin
rRNA: ribosomal RNA
TMP-SMX: trimethoprim-sulfamethoxazole

## References

[1] McNeil MM, Brown JM. The medically important aerobic *Actinomycetes*: epidemiology and microbiology. Clin Microbiol Rev. 1994;7(3):357–417. 10.1128/cmr.7.3.357 PMC358331 7923055

[2] Patel JB, Wallace Jr RJ, Brown-Elliott BA, Taylor T, Imperatrice C, Leonard DG, et al. Sequence-based identification of aerobic actinomycetes. J Clin Microbiol. 2004;42(6):2530–40. 10.1128/JCM.42.6.2530-2540.2004 PMC427891 15184431

[3] Kapadia M, Rolston KV, Han XY. Invasive *Streptomyces* infections: six cases and literature review. Am J Clin Pathol. 2007;127(4):619–24. 10.1309/QJEBXP0BCGR54L15 17369139

[4] Rose 3rd CE, Brown JM, Fisher JF. Brain abscess caused by *Streptomyces* infection following penetration trauma: case report and results of susceptibility analysis of 92 isolates of *Streptomyces* species submitted to the CDC from 2000 to 2004. J Clin Microbiol. 2008;46(2):821–3. 10.1128/JCM.01132-07 PMC2238099 18094128

[5] Weinstein MP, Murphy JR, Reller LB, Lichtenstein KA. The clinical significance of positive blood cultures: a comprehensive analysis of 500 episodes of bacteremia and fungemia in adults. II. Clinical observations with special reference to factors influencing prognosis. Rev Infect Dis. 1983;5(1):54–70. 10.1093/clinids/5.1.54 6828812

[6] Carey J, Motyl M, Perlman DC. Catheter-related bacteremia due to *Streptomyces* in a patient receiving holistic infusions. Emerg Infect Dis. 2001;7:1043–5. 10.3201/eid0706.010624 PMC2631926 11747739

[7] Ghanem G, Adachi J, Han XY, Raad I. Central venous catheter-related *Streptomyces* septic thrombosis. Infect Control Hosp Epidemiol. 2007;28:599–601. 10.1086/513619 17464923

[8] Nasher MA, Hay JR, Mahgoub ES, Gumaa SA. *In vitro* studies of antibiotic sensitivities of *Streptomyces somaliensis*—a cause of human actinomycetoma. Trans R Soc Trop Med Hyg. 1989;83:265–8. 10.1016/0035-9203(89)90675-5 2609384

[9] McNeil MM, Brown JM, Jarvis WR, Ajello L. Comparison of species distribution and antimicrobial susceptibility of aerobic *actinomycetes* from clinical specimens. Rev Infect Dis. 1990;12:778–83. 10.1093/clinids/12.5.778 2237117

[10] Kofteridis DP, Maraki S, Scoulica E, Tsioutis C, Maltezakis G, Gikas A. *Streptomyces* pneumonia in an immunocompetent patient: a case report and literature review. Diagn Microbiol Infect Dis. 2007;59(4):459–62. 10.1016/j.diagmicrobio.2007.06.009 17928185

[11] Shadzi S, Chadeganipour M. Isolation of opportunistic fungi from bronchoalveolar lavage of compromised hosts in Isfahan, Iran. Mycopathologia. 1996;133:79–83. 10.1007/BF00439117 8751826

